# Barriers and facilitators to primary care management of type 2 diabetes in Shijiazhuang City, China: a mixed methods study

**DOI:** 10.1186/s12875-024-02330-7

**Published:** 2024-03-13

**Authors:** Xuanchen Tao, Limin Mao, Puhong Zhang, Xinyan Ma, Zhenyu Liang, Kaige Sun, David Peiris

**Affiliations:** 1 The George Institute for Global Health, Beijing, China; 2 The George Institute for Global Health, Faculty of Medicine and Health, University of New South Wales, Sydney, Australia; 3 Center for Social Research in Health, University of New South Wales, Sydney, Australia; 4 Shijiazhuang Center for Disease Control, Shijiazhuang, Hebei Province China

**Keywords:** Diabetes, Primary Healthcare, China, Behavior Change Wheel, Mixed methods

## Abstract

**Background:**

The prevalence of type 2 diabetes (T2DM) in China is over 10%, affecting around 114 million people. Despite the inclusion of T2DM in the National Basic Public Health Service Program (NBPHSP), most people with T2DM experience challenges in achieving optimal management targets. This study aimed to identify barriers and facilitators of diabetes management from the perspectives of primary health care (PHC) service providers and recipients.

**Methods:**

This mixed-methods study was conducted in Shijiazhuang City, Hebei Province, China. A quantitative PHC facility assessment survey was conducted in all administrative districts and qualitative in-depth interviews were conducted in one district to government officials, medical staff, patients with T2DM, and their family members. Interviews were thematically analyzed, and all findings were synthesized using Michie’s COM-B theory.

**Results:**

A total of 197 village/community level PHC facilities and 66 township/street level PHC facilities answered the survey, and 42 in-depth interviews were conducted. The key facilitators stemmed from the NBPHSP policy, which standardized the basic infrastructure, medical equipment, and medication for the PHC facilities, provided training on NCD prevention and control, and compensated the PHC workers. However, we identified a detrimental cycle among PHC providers characterized by inadequate capacity, overwhelming workloads, insufficient income, limited career development opportunities, and challenges in attracting young talents. Although patients were covered by the national medical insurance schemes, they experienced capability constraints primarily driven by low education levels, advanced age, low health literacy, and a proliferation of misinformation. These factors influenced patients’ motivation to be actively engaged in care and contributed to inertia to intensify treatment and achieve their clinical management goals.

**Conclusion:**

This study identifies several major facilitators and barriers from the perspectives of both PHC providers and patients with T2DM. Our findings suggest there are substantial opportunities to strengthen the NBPHSP, including improving the capacity and the income level of the PHC providers, attracting and retaining skilled health workers in rural areas, supporting patients to improve their health literacy and take a more active role in their health care, and improving access to high-quality care through digital health approaches.

**Trial registration:**

ClinicalTrials.gov (record NCT02726100, 03/22/2016).

**Supplementary Information:**

The online version contains supplementary material available at 10.1186/s12875-024-02330-7.

## Background

In the new millennium, diabetes mellitus has surged to become a global health emergency [1]. China is the home to the largest number of people with diabetes in the world. Over the past three decades, the type 2 diabetes prevalence has increased from 0.7% in 1980 [2] to 5.5% in 2001 [3], 9.7% in 2008 [4], 11.6% in 2010 [5]. According to the latest published nationwide survey in 2018, about 12.4% of adults aged 18 years or older in mainland China were found to have diabetes, and 38.1% were identified as having prediabetes. Among the diabetic population, only 36.7% were aware of their diabetic condition and only 32.9% of those aware were undergoing treatment [6]. Study has projected a continuous rise in the prevalence and economic impact of diabetes in China from 2020 to 2030. The healthcare expenditure of diabetes is expected to increase from $250.2 billion to $460.4 billion from 2020 to 2030 and the per-capita economic burden of diabetes will increase from $231 to $414 per year [7]. 

In China, PHC facilities are commonly the first contact point for patients with the national health system and patients with T2DM receive most of their healthcare services in PHC facilities [8]. The PHC facilities are organized into two levels, with clinic-level facilities being managed by center-level facilities. In urban areas, community health service stations (clinic) are managed by community health service centers (center); while in rural areas, village clinics (clinic) are managed by township hospitals or health service centers (center). As part of a series of healthcare reforms, the Chinese government has made substantial investments to strengthen the PHC sector. These reforms have improved access to and affordability of PHC through increased government funding, universal health insurance coverage, and availability of essential medicines at little or no cost [9]. In 2009, China’s government promoted a package of basic public health services to strengthen healthcare delivery at the PHC level. The initial package of nine categories in 2009 had been expanded to 14 categories by 2017, among which five are relevant to diabetes management and control [10]. They are (1) residents’ health records, (2) health education, (3) healthcare to the aged population, (4) health promotion, and (5) NCD management (hypertension and T2DM). The NCD management services provide free health check, early disease detection, and regular follow-up to the target group or at-risk patients.

Despite these health reforms, there are major gaps in optimal care for people with T2DM in China. National representative surveys have shown that only 50% of treated patients with T2DM achieved the blood glucose targets [6] and only 5.6% of outpatients with T2DM met the optimal ABC targets (HbA1c < 7%, BP < 130/80 mm Hg, and total cholesterol < 4.5 mmol/L) [11]. Given that sub-optimal management targets are associated with an increased risk of diabetic complications, this will substantially increase the disease burden in years to come. Thus, it is important to understand the present challenges of diabetes management in the PHC level.

This mixed methods study aimed to identify the major barriers and facilitators to optimal diabetes care from the perspective of patients with type 2 diabetes (T2DM) and their family members, and PHC providers and health administrators.

## Methods

A mixed methods study was conducted, consisting of a PHC facility assessment and in-depth interviews with patients, family members, health care providers, and administrators.

### PHC facility assessment survey

The capacity assessment questionnaire was designed by the Chinese Center for Disease Control and Prevention (CDC) to assess the capacity of PHC facilities for the prevention and control of chronic diseases [12]. Two questionnaires were used for urban health centers and rural clinics covering the following domains: (1) general facility information; (2) finance and essential equipment; (3) training and guidelines; (4) participation in research projects (to health centers only); (5) health education activities; (6) early detection of the high-risk population; (7) severe incidents; (8) diabetes and hypertension management; (9) human resources.

The survey was administered in March 2016 in Shijiazhuang City, Hebei Province, China. We randomly selected 3 health centers and 9 clinics from each of the 22 administrative districts/counties in Shijiazhuang City. In total, we surveyed 66 centers and 197 clinics. An experienced and knowledgeable staff member from each facility was invited to answer the questionnaire.

Descriptive analysis was conducted using STATA 15.0. Means and standard deviations were reported for continuous variables with normal distribution; medians and interquartile ranges were reported when the data was skewed. Proportions were reported for categorical variables.

### In-depth interviews

For the qualitative study, we included four types of key stakeholders to explore the barriers and facilitators from the perspectives of health service providers and recipients: (1) health administrators working at CDC and health bureau (2) staff working at village clinics and township hospitals (3) patients with T2DM and (4) family members of the patients. A diverse sample of patients was selected based on age and sex data derived from the local management lists of diabetic patients. These participants were then invited to an interview along with their family members.

Four separate guides for different participant groups were initially developed by a multi-disciplinary research team (Supplementary 1). The guides were piloted and modified based on feedback. Interviews were conducted between November 2015 and June 2016 at Luquan - a suburban district with both urban and rural PHC facilities - in Shijiazhuang City, Hebei Province, China. Interviews were conducted face-to-face by the research team in a private environment to ensure participant confidentiality. All interviews were audio-recorded and transcribed verbatim in Chinese.

The demographic information of participants was tabulated. For thematic analysis, researchers met regularly as interviews were conducted to agree on emerging themes, determine the focus for subsequent interviews and assess when thematic saturation was being achieved. From the initial coding and discussion of interview transcripts, a hierarchical coding tree was first developed and iteratively refined as more transcripts were analysed (Supplementary 2). Two researchers independently coded the interview transcripts using NVivo 11, compared their results, and resolved discrepancies through discussions.

### Mixed methods integration of survey and interview data

**Fig. 1:**
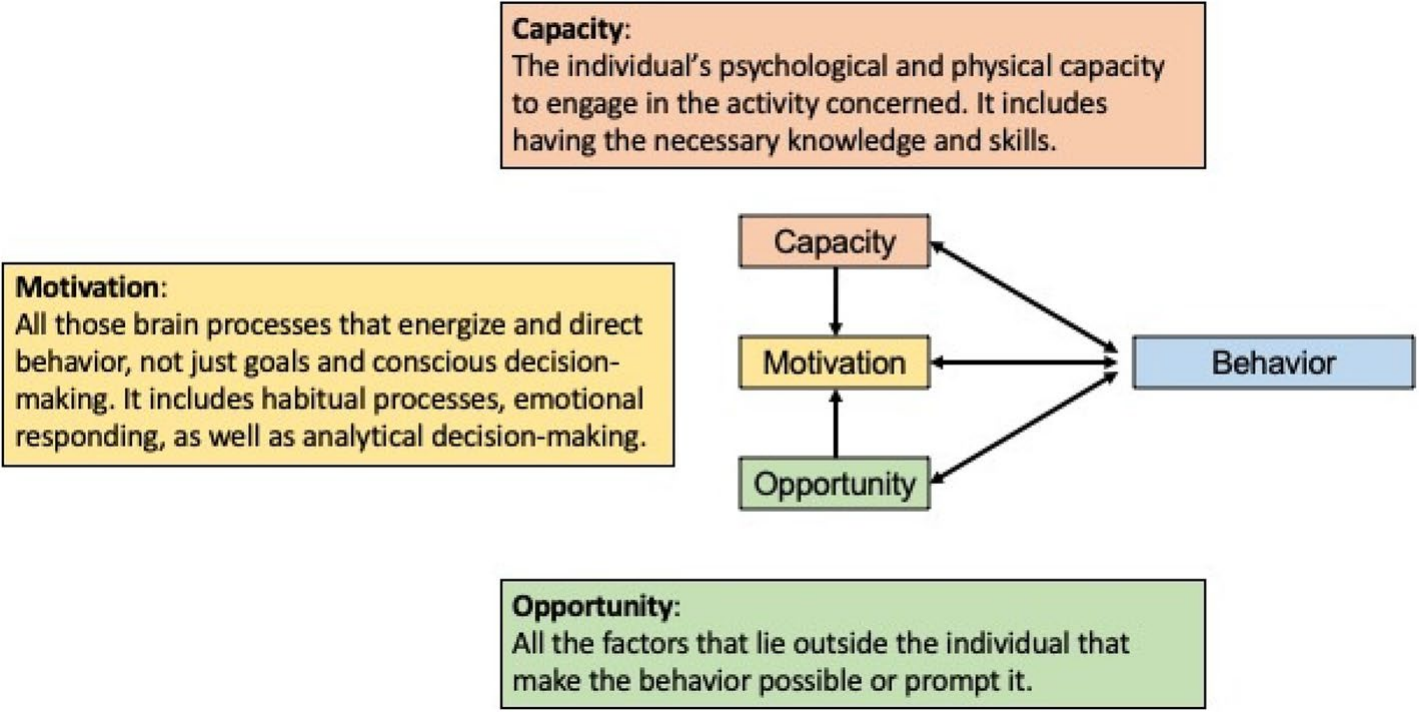
The Capability, Opportunity, and Motivation Behavior (COM-B) model from the Behavior Change Wheel (BCW) theory

The Capability, Opportunity, and Motivation Behavior (COM-B) model from the Behavior Change Wheel (BCW) was used to integrate the qualitative and quantitative components of this study [13]. The COM-B model, encompassing capability, opportunity, and motivation as key constructs, provided a systematic framework for understanding the behavior system (See Fig. 1). The COM-B model suggests that for behavior change to occur, there needs to be an interaction between these three components. Understanding the interplay of capability, opportunity, and motivation can help design effective interventions to promote or modify behaviors. Findings from the facility assessment and key themes identified from the qualitative study were classified into these three components and then labelled as either barriers or facilitators.

## Results

### Quantitative findings

#### PHC facilities

We surveyed 66 health centers (13 urban and 53 rural) and 197 clinics (34 urban and 163 rural). The median numbers of health staff in each facility were 11.5 (Interquartile range, IQR: 5–59), 2 (IQR: 1–4), 37 (IQR: 32–48), 28 (IQR: 18–36) for urban clinics, rural clinics, urban health centers, and rural health centers, with 1.3 (IQR: 0.8-2.0), 0.7 (IQR: 0.5-1.0), 2 (IQR: 1.3–3.6) and 1 (IQR: 0.8–1.8) full-time equivalent (FTE) staff involved in hypertension and diabetes management, respectively (Table 1). Nealy all the clinics were equipped with basic equipment such as blood pressure measuring devices, glucometers, electrocardiographs, and audiovisual equipment. Advanced equipment such as biochemical analyzers, ultrasound machines, and X-ray machines was more available in center-level PHC facilities. 95% of all PHC facilities were provided training for NCD prevention and control.

**Table 1:** Assessment survey of PHC facilities

	PHC clinics		PHC centers	
Characteristics	Urban (*N* = 34)	Rural (*N* = 163)	Urban (*N* = 13)	Rural (*N* = 53)
**Service coverage, median (IQR)**				
Area (km^2)	2.0 (0.4–2.5)	2.3 (1.2-4.0)	3.1 (1.7–7.2)	48.1 (25.0-69.9)
Permanent residents	8158 (6416–11,000)	1980 (1054–3150)	32,761 (23,278–34,572)	33,486 (24,430–46,630)
Population aged over 65	820 (540–1477)	180 (102–311)	2712 (2200–5121)	2966 (2120–4668)
**Human resources per each PHC facility, median (IQR)**				
Total staff	11.5 (8–14)	2 (1–4)	37 (32–48)	28 (18–36)
Licensed doctor and assistant doctor^a^	5 (4–7)	1 (0–1)	18 (13–22)	11 (7–19)
Registered nurse	4 (2–6)	0 (0–0)	11 (10–13)	4 (2–6)
Others	1 (0–3)	1 (1–3)	8 (6–17)	11 (5–16)
**Human resources per 1,000 residents, median (IQR)**				
Total staff	1.4 (0.9–2.2)	1.4 (0.9–1.9)	1.4 (1.1–2.1)	0.9 (0.6–1.2)
Licensed doctor and assistant doctor^a^	0.8 (0.5–1.1)	0.4 (0-0.8)	0.6 (0.5–0.9)	0.4 (0.3–0.6)
Registered nurse	0.5 (0.3–0.8)	0 (0–0)	0.4 (0.3–0.5)	0.1 (0.1–0.2)
Others	0.1 (0-0.3)	0.8 (0.3–1.4)	0.4 (0.2–0.5)	0.3 (0.2–0.4)
**Staff involved in NCD prevention and control works** ^**b**^, **median (IQR)**				
Number of staff involved per facility	3 (2–4)	2 (1–2)	5 (3–6)	2 (1–3)
Cumulative full-time equivalent staff per facility	1.3 (0.8-2.0)	0.7 (0.5-1.0)	2 (1.3–3.6)	1 (0.8–1.8)
**Equipment availability, %**				
Blood pressure measuring devices	100	98.8	100	98.1
Glucometer	97.0	99.4	100	98.1
Electrocardiograph	100	83.4	100	98.1
Audiovisual equipment for health education	91.2	64.4	84.6	100
Biochemical analyzer	32.3	0.6	100	96.2
Ultrasound machine	17.7	3.1	100	98.1
X-ray machine	2.9	1.8	53.9	86.8
**Trainings of NCD prevention and control**				
Trainings received (yes), %	100	96.3	100	88.7
Trainings received in last year (person-time), median (IQR)	8.5 (6–15)	9 (4–16)	25 (12–39)	8 (4–17)
Ever received on-site technical guidance from superior institutes (yes), %	94.1	94.4	-	-
Ever provided on-site technical guidance to inferior institutes (yes), %	-	-	77.0	94.3
Ever received supports from hospital experts (yes), %	58.8	61.5	92.3	73.1
**Hypertension management reported by PHC provider, median (IQR)**				
Number of registered patients	465.5 (353–707)	190 (97–299)	-	-
Number of patients followed-up	461.5 (350–808)	178 (96–283)	-	-
Proportion of patients who proactively seek PHC services (yes), %	41 (20–57)	65 (40–81)	-	-
Proportion of patients who own BP measurement devices at home (yes), %	80 (30–89)	20 (10–40)	-	-
**Diabetes management reported by PHC provider, median (IQR)**				
Number of registered patients	183 (145–303)	50 (26–95)	-	-
Number of patients followed-up	171 (130–263)	50 (25–86)	-	-
Proportion of patients who proactively seek PHC services (yes), %	49 (21–70)	65 (36–82)	-	-
Proportion of patients who own glucometers at home (yes), %	50 (19–70)	10 (5–30)	-	-

*NCD *Non-communicable chronic disease, *PHC *Primary health care, *IQR *Interquartile range

^a^Completion of medical college is required to become a licensed doctor and junior medical college training is required to become a licensed assistant doctor. Meanwhile, they also need to pass the National Practicing Doctor (or Assistant Doctor) Examination and periodic government assessments [8]

^b^NCD prevention and control works are mainly the hypertension and diabetes management as per the NBPHSP

In urban areas, the proportion of hypertensive patients who proactively seek PHC services was 41% (IQR: 20-57%), while in rural areas, the proportion was higher at 65% (IQR: 40-81%). Similarly, among diabetic patients, 49% (IQR: 21-70%) in urban areas and 65% (IQR: 36-82%) in rural areas were found to be actively seeking PHC services. For each urban clinic, around 550 patients (465.5 (IQR: 353–707) hypertensive patients and 183 (IQR:145–303) diabetic patients) were registered and managed by 1.3 FTE, while for each rural clinic, around 240 patients (190 (IQR:97–299) hypertensives and 50 (IQR:26–95) diabetics] were registered and mostly managed by 0.7 FTE. Only around half of the patients proactively seek PHC services in both urban and rural areas. In urban areas, approximately 80% (IQR:30-89%) of hypertensive patients and 50% (IQR:19-70%) of diabetic patients owned self-monitoring devices at home. In contrast, in rural areas, the ownership of self-monitoring devices was significantly lower, with only 20% (IQR:10-40%) of hypertensive patients and 10% (IQR:5-30%) of diabetic patients having such devices at home.

### PHC providers working in chronic diseases prevention and control

The facility assessment survey investigated the key characteristics of PHC providers who were involved in NCD prevention and control (Table 2). The mean age of these PHC providers in centers and urban clinics was 37 years, whereas the mean age of providers in rural clinics was 47 years. Healthcare providers at rural clinics had the lowest education level (72.6% had a degree in technical school or below). More than 90% of healthcare providers in urban areas had a license to practice. However, only 47.9% of healthcare providers in rural clinics and 75.2% of healthcare providers in rural centers had a license. Most of them had a salary between 1500 and 3000 CNY, but more than half (61.9%) of the PHC providers in rural clinics earned less than 1500 CNY per month.

**Table 2:** Assessment survey of staff involved in NCD prevention and control at PHC facilities

	Health clinics		Health centers	
Characteristics	Urban (*N* = 107)	Rural (*N* = 335)	Urban (*N* = 58)	Rural (*N* = 122)
Age (year), mean (SD)	37.0 (9.7)	46.9 (11.7)	37.3 (8.9)	37.0 (8.1)
Female, %	74.0	32.9	75.9	58.2
Education, %^a^				
Technical school or below	11.9	72.6	5.2	25.4
Junior college	62.4	25.2	44.8	60.7
College or higher	25.7	2.3	50.0	13.9
Major, %				
Public health	3.0	6.9	1.7	5.0
Clinical medicine	53.5	69.6	72.4	56.7
Other medical majors	40.6	13.9	25.9	31.7
Else	3.0	9.6	0.0	6.7
Staff with licenses, %^b^	93.1	47.9	92.9	75.2
Salary per month, %				
< 1500 CNY	5.0	61.9	11.1	22.1
1500–3000 CNY	78.2	35.7	83.3	76.2
> 3000 CNY	16.8	2.4	5.6	1.6

^a^Technical School: 3 years of medical education after 9 years of primary and secondary education; Junior medical college: 3 years of medical education after 12 years of primary and secondary education; Medical college: 5 years of medical education after 12 years of primary and secondary education

^b^Completion of medical college is required to become a licensed doctor and junior medical college training is required to become a licensed assistant doctor. Meanwhile, they also need to pass the National Practicing Doctor (or Assistant Doctor) Examination and periodic government assessments [8]

### Qualitative findings

A total of 42 participants (4 officials, 7 medical staff, 18 patients with T2DM, and 13 family members) were interviewed. The interviews lasted from 0.5 to 1.5 h for officials and medical staff, and from 10 min to 0.5 h for patients and their family members. The detailed demographic information of the interviewees was summarized in Supplementary 3.

### Capacity of patients with T2DM

#### Insufficient health literacy of the patient population

The health literacy level of patients was generally low, and their knowledge of diabetes management tended to be either absent or mistaken and easily influenced by factually dubious information. More than half of the interviewed patients with T2DM were over 50 years old and their education levels were only high school or below. It was common that patients without severe symptoms or complications tended to not perceive blood glucose and blood pressure management as a health priority. They perceived themselves as healthy and stopped taking medication when they felt “well” (absence of sick symptoms). Few patients understood the significance of regular monitoring and the need for long-term blood glucose control to prevent complications.

*“I take my pills twice a day. Sometimes I stop taking them if I feel “well””. (patient-female-61-below primary school)*

In terms of managing modifiable lifestyle risk factors, the most mentioned barriers were unhealthy diet and physical inactivity. The typical understanding of diet control is to avoid eating sweet food and sugar, without considering the overall energy intake. Though patients tended to control their alcohol and tobacco use when feeling unwell, many expresses challenges in long-term abstinence.

*“If my blood glucose is raising – I can tell by feeling unwell – I do not use a glucometer; I just drink less (alcohol) and that is all.” (patient-male-66-middle school)*.

It was also common that patients prioritized expensive health supplements or traditional Chinese medicine over comparably affordable oral anti-diabetic drugs. Some were convinced by misleading TV advertisements and their relatives, and others complained about the side effects caused by Western medicine.

*“Sometimes, villagers prefer to listen to drug salesmen or audio/TV advertisements (over us).” (village doctor-male-45-technical school)*.

*“I watched the TV commercial about bee propolis treating diabetes. I have been taking propolis for 3 years to treat my diabetes. It costs me about 2000 CNY per year.” (patient-female-51-middle school)*.

*“My father stopped using insulin one year ago; we go to Shanxi Province to get medicine from traditional Chinese doctors, and it costs us 1000 CNY per month; my father’s blood glucose is still uncontrolled.” (family member-male-27-technical school)*.

All patients knew they were covered by urban and rural resident medical insurance. Their most direct experience was from the reimbursement of in-patient care at hospitals, while only a few patients understood the reimbursement scheme of outpatient care at PHC facilities.

*“I do not understand this (reimbursement scheme). I get what I get (at the outpatient care).” (patient-female-65-below primary school)*.

### Health education activities organized by PHC facilities

PHC facilities organized various regular health education activities, including banner displays, free medical consultations, lectures, distribution of pamphlets, and broadcasting. However, these activities often reached individuals who were already aware of the significance of the disease, while struggling to effectively reach those who were most in need of awareness and intervention due to their high-risk profiles. Furthermore, there was a notable absence of incentives and evaluation for these health education activities.

*“To be honest, not so many people come to health education activities. Some people might come, while others don’t even come when called. Those who are aware of the importance always show up, but those who are busy earning money and think it doesn’t matter, they don’t come.” (village doctor-male-47-technical school)*.

### Opportunity for patients with T2DM

#### The National Basic Public Health Service Program and health insurance

Under the NBPHSP, patients were provided with a diverse range of healthcare services. While patients demonstrated active participation in free health examinations, and free BP/BG follow-up measurements, their engagement in other services such as health education services was relatively lower.

*“Villagers started participating in the health examination since 2007 or 2008. Compare now and then, you could feel that patients have paid significantly more attention to their health. For residents aged over 35, we provide free health examinations once per two years.” (official-female-31-bachelor)*.

The health education and health promotion services reach the public through various activities, including bulletin boards, public health consultation, seminar, and individualized health education. The education content covers health literacy, lifestyle risk factors, and chronic disease information including hypertension, diabetes, and cardiovascular diseases. In addition, every village health clinic is required to have a designated room for health education.

*“Some (health education) was done by the township health centers and others were done by village clinics…in terms of the education content, we have detailed health-promoting material every year, as well as health education prescriptions targeting at-risk populations.” (official-female-31-bachelor)*.

All the interviewed patients were insured. Each villager paid 110 CNY insurance premium annually and the government subsidized for another 380 CNY. The reimbursement rate for inpatient care was higher compared to outpatient care, and it was also typically higher for reimbursement at lower-level PHC facilities.

*“I get a 50% discount for Metformin.” (patient- male-53-primary school)*.

### Support from family members and mhealth technology

Most of the interviewed family members expressed their willingness to take care of the patients with T2DM. However, their knowledge of T2DM management was also limited. Meanwhile, the patients hesitated to seek assistance from their busy working daughters or sons, as they didn’t want to impose any additional burden on them. In contrast, patients felt more comfortable seeking help from their spouses.

*“My sons and my daughters have jobs. They are all busy. Sometimes, they come home from work when I’m already in bed, and they leave for work before I even wake up.…I will ask my husband to drive me to the county hospital when I feel sick.” (patient-female-70-middle school)*.

All of the officials, medical staff, and family members we interviewed reported owning a smartphone, and several family members specifically mentioned having prior experience with mobile health management applications (Supplementary 2).

### Motivation of patients with T2DM

#### Limited motivation to engage in self-monitoring and medication adherence

Few patients knew about early symptoms of diabetes complications, or their subsequent harm and potential for catastrophic health expenditure. Due to this lack of awareness regarding the severity of complications associated with diabetes, there existed a diminished motivation to proactively manage and control their respective health conditions. Some patients owned glucometers or blood pressure measuring devices at home, but few monitored and tracked health themselves regularly. Some only used the measuring devices when they felt unwell. Some patients reported that they stopped taking medication when the symptoms improved.

*“I do not think my hypertension is a huge problem…I stopped taking my pills…my daughter bought me a glucometer, but I do not use it.” (patient-female-49-middle school)*.

Although most patients actively participated in the free annual health examination and the face-to-face follow-ups provided by the NBPHSP, the management relationship between PHC providers and patients remained passive. Few patients paid enough attention to their health condition, or are self-motivated to make healthy behaviour changes unless their diseases develop into more serious symptoms or complications. Village doctors found it difficult to organize health education or examination events and relied on incentives such as gifts to promote attendance.

*“The majority of patients only pay attention to their health conditions when we ask them to. The policy demands us to do so. (official-female-31-bachelor) Half of my patients do not listen to me because of their low health awareness… if patients do not pay attention to their own health, nothing will work”. (village doctor-male-47-technical school)*

### Treatment inertia

**Fig. 2:**
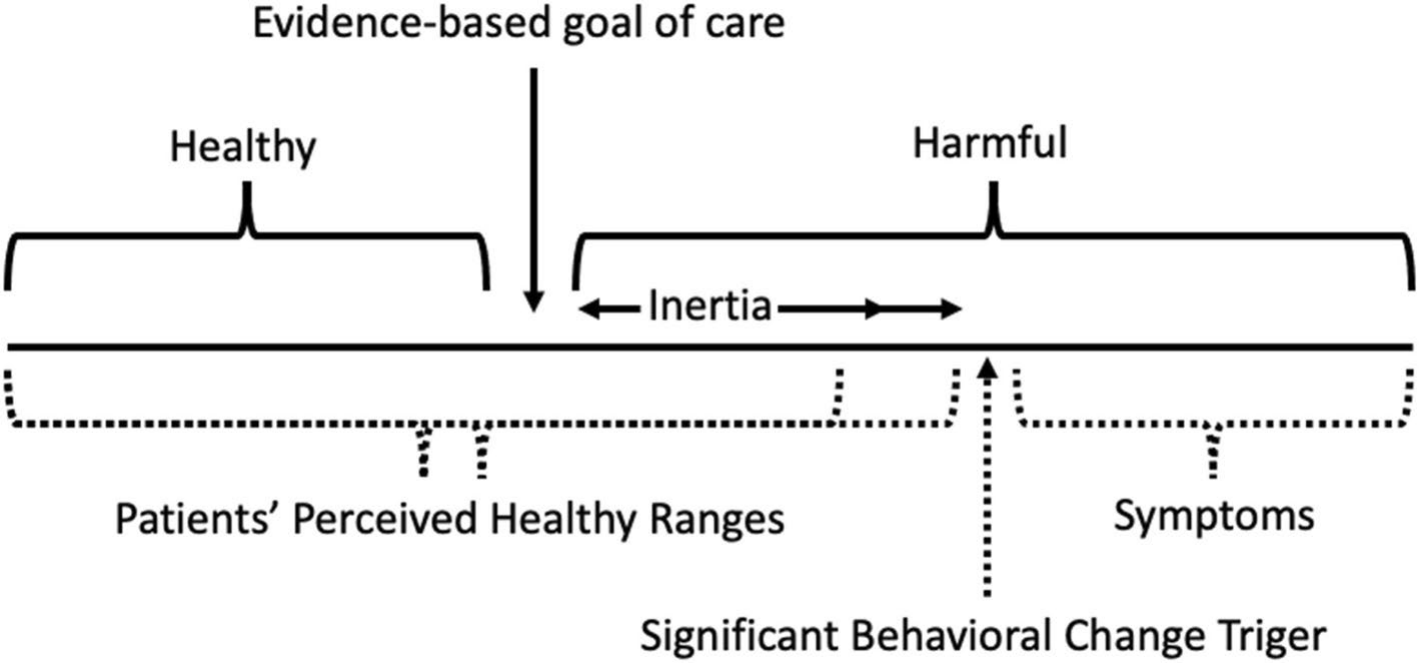
Diagram of patient’s inertia

Our study found a lack of interest in treatment intensification from patients and doctors when patients were not at evidence-based goals for care. Again, patient motivation to change management was often driven by the presence of symptoms of feeling unwell. Patients tended to consider themselves healthy as long as they ‘feel well’, even when their blood pressure or blood glucose was uncontrolled. There were also substantial gaps between the perceived healthy range of patients and the evidence-based goal of care (see Fig. 2). Similar gaps in perceptions of optimal care were observed in interviews with healthcare providers.

*The doctor told me to take anti-diabetes drugs, but I stopped taking my pills when I felt ok. Then, my blood glucose raised again. (patient-female-65-illteracy)*

*If my fasting blood glucose is not over 8.0 mmol/L, I do not need any medication. (patient-male-65-middle school)*

Doctors were reluctant to intensify the treatment as they worried about the negative feedback (experience of discomfort) from the patients and safety issues with the elderly patients.

*“Some village doctors are not capable enough (to control the patients’ chronic disease conditions).” (county doctor-male-40-junior college)*.

### Capacity of Healthcare Providers

The local government provides a series of themed training based on the NBPHSP to village doctors twice per year. The township health centers also actively provide regular training to the village clinics. However, village doctors complained that the training was time-consuming and overwhelming.

*“We provide training to village doctors twice a year. Each year, there is a test for the training. Village doctors need to pass the test to practice… we train village doctors according to the code of the national basic health services.” (official-female-31-bachelor)*.

*“I received training about 7 or 8 times per year (from different institutes).” (village doctor-female-45-technical school)*.

### Opportunity for Healthcare Providers

#### PHC facility infrastructure

In the studied district, there are clear policy documents that aim to integrate local healthcare resources, increase health services coverage, and standardize the infrastructure of village clinics.

#### Access to essential medicine

There is a designated pharmaceutical room in every village clinic. The government policy document requires every clinic to use medicine in the Nation List of Essential Medicine or Supplementary Provincial List of Essential Medicine. Village clinics regularly report their demand for medication quantity to township health centers and township health centers make all the procurements centrally.

### Health information systems

In our studied district, all healthcare providers had Internet and computer access. Village doctors primarily used the information system to (1) create residents’ health records, (2) track patients’ follow-ups and (3) upload the results of annual health checks. Township doctors validated village doctors’ work via the system, which local officials used for statistics and reports. Some doctors complained about repetitive, tedious data entry, lack of integration with hospital systems, and inability to share data across different levels of facilities.

#### Motivation of Healthcare Providers

##### Low income, heavy workload and unclear career development path

Village doctors complained about their low income and the income gap between rural and urban areas. Some doctors ceased practice because of financial difficulties. There were two major income sources for doctors – diagnosis and treatment fees and the NBPHSP subsidy. The amount of subsidy depended on the amount of public health work done, such as the number of hypertensive patients being followed up. Meanwhile, village doctors faced a heavy workload of managing patients with hypertension and diabetes. Their main responsibilities in hypertension and diabetes prevention and control include (1) conducting population screening (2) establishing and maintaining health records (3) providing optimal treatments (4) conducting at least four follow-up visits per year and updating patients’ health records. However, NCD management is just a small part of the 14 responsibilities required by the NBPHSP. In addition, there were limited opportunities for promotion, specialization, or career advancement within the rural healthcare system. This lack of clear career pathways can be demotivating and hinder professional growth.

*“Our village doctors don’t earn much…they hardly make a living (if their solo income is from being a village doctor) …the financial issue is definitely a constraint.” (official-female-31-bachelor)*.

#### Recognition in society and job fulfillment

Recognition of social status and relationship with patients was associated with the job satisfaction of the village doctors. Most of our interviewed patients were satisfied with the healthcare services and showed respect to their local doctors.

*“I’m quite satisfied (with my village doctor). I have been seeking medical treatment here ever since my childhood.” (family member-female-18-technical school)*.

*“The capability of the doctor and his prestige in the village matter a lot…if the patients respect the doctor, they will listen to whatever he says…if not, they will just disobey.” (official-female-31-bachelor)*.

### Summary of the quantitative and qualitative findings

Bringing the quantitative and qualitative findings together, we identified multiple barriers and facilitators of diabetes management (Table 3).

**Table 3:** Summary of the barriers and facilitators of diabetes management at PHC level

COM-B Component	Barrier or Facilitator	Quantitative Findings	Qualitative Findings
**Capacity (PHC service providers)**	**Barrier 1: Education gap**	Education level is low in general. In urban areas, less than half of the PHC providers hold a college degree: 25.7% in clinics, 50.0% in centers. In rural areas, the figures are only 2.3% and 13.9%, respectively.	At rural clinics, most village doctors only attended technical school. Retaining doctors with higher education levels or experience working at hospitals in rural clinics posed a significant challenge due to low income, heavy workload, and unclear career development.
	**Barrier 2: Licensure gap**	More than 90% doctors in urban PHC facilities were licensed, whereas fewer doctors were licensed in rural areas (47.9% in clinics and 75.2% in centers).	Despite the legal prohibition on unlicensed practice, this study has observed the presence of village doctors practicing without a license.
	**Barrier 3: Ageing of doctors in rural clinics**	The mean age of village doctors, at 47 years, was notably higher compared to the mean age of 37 years observed among other PHC facilities. About 19.1% village doctors were older than 60 years vs. 2.6% doctors in other PHC facilities.	The village doctors we interviewed had an average age of 43 years. It was challenging to recruit young and capable doctors in remote areas.
	**Barrier 4: Overburdened**	Not including other public health services, around 400 patients with hypertension or diabetes need to be managed using one FTE by medical staff. This is a tremendous workload for NCD managers considering at least 4 BP/BG measurements and face-to-face consultations should be provided for each of the patients.	In rural and urban clinics, except for clinical services, more than 14 public health services should be provided by PHC providers. In rural area, half of their time has to be devoted to NCD management.
	**Barrier 5: Clinical inertia**		Doctors were reluctant to intensify treatment to the elderly patients who were not at evidence-based goals for care. They were worried about the negative feedback from patients, and they were not confident about their capacity.
	**Facilitator 1: Workshops on NCD prevention and control**	Almost all PHC facilities (95%) reported that they received training workshops about NCD prevention and control which facilitated the implementation of NBPHSP.	NCD prevention and control trainings were provided by local health department, CDC, hospitals, center level PHC facilities and other institutes.
	**Facilitator 2: On-site technical guidance and visits by hospital chief experts**	Most the clinic level PHC facilities (94%) reported that they have *received* on-site technical guidance provided by the center level facilities, while 77.0% urban centers and 94.3% rural centers reported that they have *offered* such guidance to clinic level facilities. The proportions of PHC facilities that received hospital expert visits were 58.8%, 61.5%, 92.3%, and 73.1% for urban clinics, rural clinics, urban centers, and rural centers respectively.	Hospital medical experts from specific fields visit PHC facilities to offer consultation services, guidance, and support.
**Capacity (PHC service recipients)**	**Barrier 1: Low education level and ageing population**		More than half of the interviewed patients with T2DM were over 50 years old and all of their education levels were high school or below.
	**Barrier 2: Insufficient health literacy and misinformation**		Patients’ knowledge of diabetes management was often lacking, incorrect, or easily misled by misinformation. Misleading TV advertisements and quack doctors promoting the less effective and expensive treatment regimes.
	**Facilitator 1: Health education activities organized by PHC facilities**	The availability of audiovisual equipment for health education purposes is as follows: 91.2% in urban clinics, 64.4% in rural clinics, 84.6% in urban centers, and 100% in rural centers. 95% clinics have organized health education lectures on NCD prevention and control. 98% clinics have distributed promotional materials.	The health education activities included (1) banner (2) free medical consultation (3) lecture (4) pamphlets (5) broadcasting. However, most patients demonstrated passive engagement in these activities, and there was a lack of incentives and evaluation for these activities.
**Opportunity (PHC service providers)**	**Facilitator 1: High availability of essential medical equipment across all levels of PHC facilities and access to advanced medical equipment at center level PHC facilities**	99% PHC facilities were equipped with blood pressure measuring devices and glucometers; 89% owned an electrocardiograph and 76% owned audiovisual equipment for health education purposes. 98% health centers were equipped with ultrasound machines, 97% had a biochemical analyzer, and 80% had an X-ray machine (54% in rural areas and 87% in urban areas).	There were clear policy guidelines in place to regulate the establishment of PHC facility infrastructure, taking into account the size of the population it served.
	**Facilitator 2: Access to essential medicine**		Affordable drugs are available in PHC level based on the National List of Essential Medicines and the Provincial List of Essential Medicines.
	**Facilitator 3: Health information systems**	The majority of patients’ information was digitally archived and regularly updated.	All PHC facilities had access to computers and internet. The health information system was established to streamline the management and follow-up of NCD patients. However, the PHC system was segregated from the hospital system which caused repetitive data entry work and discontinuity in care.
**Opportunity (PHC service recipients)**	**Barrier 1: Lack of self-monitoring**	It was estimated that 80% of hypertensive patients in urban area owned blood pressure measuring devices at home, whereas only 20% rural patients owned such equipment. 50% urban patients owned glucometers at home, and only 10% rural patients had glucometers.	Some patients owned glucometers or blood pressure measuring devices at home, but few monitored and tracked health themselves regularly. Some only used the measuring devices when they felt unwell.
	**Facilitator 1: The NBPHSP and health insurance**	On average, each urban clinic followed up 461.5 hypertensive patients and 171 diabetic patients. Each rural clinic followed up 178 hypertensive patients and 50 diabetic patients. The NBPHSP required village doctors to conduct at least 4 BP/BG measurements and face-to-face consultations to them per year.	The NBPHSP provided of a standardized set of health services such as establishing health records, diabetes and hypertension screening, follow-up, and health check. All interviewed participants were covered by health insurance and experienced reimbursement.
	**Facilitator 2: Support from family members and mHealth technology**		Family members expressed their willingness to take care of the patients, but their knowledge on diabetes management was inadequate. Several family members mentioned their previous experience with mobile health management applications.
**Motivation (PHC service providers)**	**Barrier 1: Pay gap**	A significant proportion of village doctors (61.9%) received a salary below 1500 CNY, whereas the majority of PHC providers in other facilities were paid between 1500 and 3000 CNY (78.2% in urban clinics, 83.3% in urban centers, and 76.2% in urban clinics).	Village doctors complained about the low income and inadequate social benefits. PHC providers were compensated based on the quantity of public health work they done, such as the number of hypertensive patients they followed up, rather than the quality of the services they delivered.
	**Barrier 2: Heavy workload and unclear career development path**	In regard to NCD management and control, PHC providers were tasked with establishing health records for patients, conducting at least four follow-ups per year, and participating in intensive trainings. Given the size of the patient population and the limited number of PHC staff, these responsibilities place a substantial burden on PHC doctors.	NCD prevention and control represents only a fraction of the 14 tasks mandated by the NBPHSP. There were limited opportunities for promotion, specialization, or career advancement within the rural healthcare system.
	**Facilitator 1: Recognition in society and job fulfillment**		Job satisfaction among village doctors is positively influenced by factors such as recognition of social status and maintaining good relationships with patients.
**Motivation (PHC service recipients)**	**Barrier 1: Lack of motivation**	A limited proportion of patients were reported to actively seek health services from PHC facilities (41% hypertensive patients in urban clinics, 65% hypertensive patients in rural clinics, 49% diabetic patients in urban clinic and 65% diabatic patients in rural clinics)	Patients didn’t acknowledge the seriousness of diabetes complications and they were not motivated to monitor themselves or follow the doctors’ prescription unless they felt the physical discomfort. Doctors had to urge and push patients to participate in community health education events.
	**Barrier 2: Patient inertia**		A few patients did not believe that their treatment had to be intensified or continued unless they noticed physical symptoms.

## Discussion

This mixed methods study identified key barriers and facilitators of diabetes care from the perspectives of PHC service providers and service recipients. PHC providers face challenges due to a lack of capacity, including gaps in education and licensure, an aging profession, high workloads, and clinical inertia. Motivational factors such as pay gaps, burnout and unclear career paths were also identified as key barriers. Similarly, patients experience capacity constraints primarily driven by low education levels, advanced aging, insufficient health literacy, and a proliferation of misinformation. These factors influenced patient motivation to engage in care and contributed to inertia to intensify treatment and achieve clinical management goals.

There are major gaps in optimal care for people with T2DM in China. National representative surveys have shown that only 50% of treated patients with T2DM achieved the blood glucose targets, [6] and only 5.6% of outpatients with T2DM met the optimal ABC targets (HbA1c < 7%, BP < 130/80 mm Hg, and total cholesterol < 4.5 mmol/L) [11]. Given that sub-optimal management targets are associated with an increased risk of diabetic complications, this will substantially increase the disease burden in years to come. Although several studies have investigated the barriers or facilitators associated with diabetes management in China, they have tended to focus exclusively on either patient self-management [14, 15] or provider service delivery factors [16], and few have combined patient and provider perspectives together.

Most patients with T2DM in this study were older with low education levels, especially in rural areas. These demographic factors may influence the capacity to retain information, diabetes knowledge, and disease self-management skills [13]. Although education levels are improving, the issue of an ageing population will remain a major challenge to the public health system in China [17]. A lack of knowledge about diabetes self-management has been reported as a major barrier in previous studies [18–20]. In our study, we found that patients’ knowledge about diabetes management was often missing (e.g., low awareness of diabetes complications), mistaken (e.g., intermitted medication use driven by the presence of symptoms), and easily misled (e.g., belief in health advice from quack doctors and false information in television advertisements). Although both doctors and patients considered self-monitoring of blood glucose important, most patients rarely did this consistently [21]. Similar to the previous study, we also found that temporary improvements in glycaemic management could lead to discontinuity of medication use [22]. Health education activities organized by PHC facilities are an important mechanism to enhance patient awareness and knowledge of T2DM. However, such activities tend to only reach people who are already aware of the importance and are less effective in reaching others.

The NBPHSP itself, comprehensive health insurance schemes, family support and mHealth technology were identified as major ‘opportunity’ facilitators for patients. The NBPHSP has been expanded and plays an important role in providing universal health coverage in China [23] with over 95% of China’s population covered by basic medical insurance [24]. Family support was identified as a facilitator and this finding is consistent with previous studies [19, 25, 26]. However, family member knowledge of diabetes management is also limited and requires greater support from medical authorities. mHealth, defined as “medical and public health practice supported by mobile devices” [27], has been shown to improve diabetes management, hypertension management, weight loss, physical activity, attendance rate, and adherence to treatment [28]. A recent large-scale Chinese study found that an mHealth-enabled diabetes management intervention improved control in primary care settings [29]. Given the widespread smartphone ownership in this study, a mobile application could assist PHC doctors and family members to bridge the knowledge gap, improve communication with healthcare providers, and promote better self-management.

Patients were most motivated to make a change when they felt ‘unwell’ and tended to not consider regular self-monitoring as a priority. Consistent with prior research, our findings highlight a limited awareness of the potential health harm and financial risks of diabetes complications, which in turn undermines patients’ motivation to actively participate in self-management [22]. Finally, patients tended to have a passive management relationship with care providers which undermines the effectiveness of various health interventions provided by the PHC facilities, a finding also consistent with previous studies [19, 25, 26]. 

‘Capacity’ facilitators for healthcare providers included workshops on NCD prevention and control, on-site technical guidance, and visits by hospital chief experts. A wide range of interventions recommended by the World Health Organization have been implemented in China to attract and retain health professionals, including medical education, financial incentives, regulation, and professional development [30]. However, the levels of education and qualification among PHC professionals in rural areas remain low and it is a challenge to recruit young and capable doctors to work in remote areas [31, 32]. ‘Opportunity’ facilitators for healthcare providers include clear policy guidelines for PHC facility equipment and infrastructure, internet access in the facility, and a consistent supply of essential medicines. Conversely, a major ‘opportunity’ barrier related to siloed health information systems with PHC facility information not integrated with hospital information systems. This caused problems such as duplicate data entry work and discontinuity in care [32]. The two main ‘motivation’ barriers for care providers were inadequate remuneration and minimal incentives to attract young and capable doctors to rural and remote areas. Healthcare professionals experienced burnout, spending much of their time on chronic disease management which led to opportunity costs in fulfilling other tasks required by the NBPHSP. The turnover intention among rural PHC providers in China is as high as 44.1%, two to four times greater than in high-income countries [33]. The main ‘motivation’ facilitator was a strong patient-doctor relationship which enhanced doctor prestige and fostered patient trust.

## Strengths and limitations

The mixed method design allowed triangulation between quantitative and qualitative data sources which added greater depth to overall study findings than each component in isolation. The PHC facility survey was a random sample which enhanced representativeness. The qualitative interviews involved a diverse range of stakeholders, to generate a comprehensive understanding of care facilitators and barriers from multiple perspectives. However, this study has several limitations. Firstly, this study was conducted in one province of China, although it is broadly similar to other provinces in middle eastern China, the results cannot fully represent other parts of China. Secondly, it’s essential to note that the interviews were conducted exclusively in one district, and therefore, the outcomes may not be universally applicable to other districts characterized by distinct demographics and health system dynamics. However, it’s noteworthy that Luquan, the chosen district, encompasses both urban and rural settings, providing a comprehensive representation of diverse environments within its suburban scope.

## Conclusions

This mixed methods study identified key barriers and facilitators to diabetes management at the PHC level from the perspectives of patients with T2DM and their family members, and healthcare providers and health administrators in China. We recommend greater attention be paid to workforce strengthening policies, particularly in rural areas; and strategies to enhance patient capability and motivation to proactively engage in self-management and care provision. We also recommend future research focus on developing and evaluating innovative strategies to enhance uptake of the NBPHSP such as provider and patient-facing mHealth interventions.

### Supplementary Information

**Supplementary Material 1.**

## Data Availability

The datasets used during the current study are available from the corresponding author upon reasonable request.

## Abbreviations

T2DM: Type 2 Diabetes Mellitus
PHC: Primary Healthcare
NBPHSP: The National Basic Public Health Service Program
NCD: Non-communicable Diseases
CDC: Center for Disease Control and Prevention
COM-B: Capability, Opportunity, Motivation and Behavior
FTE: Full-time equivalent
IQR: Interquartile Range
CNY: Chinese Yuan
TV: Television
BP/BG: Blood Pressure and Blood Glucose
