# Supplementary material for: Barriers and facilitators to primary care management of type 2 diabetes in Shijiazhuang City, China: a mixed methods study
Source: BMC Prim Care. 2024 Mar 13;25:84. doi: 10.1186/s12875-024-02330-7 (PMC10935988; doi:10.1186/s12875-024-02330-7)
Supplement: Supplementary file 1 — Supplementary Material 1. [file 12875_2024_2330_MOESM1_ESM.docx]

Barriers and Facilitators to Primary Care Management of Type 2 Diabetes in Shijiazhuang City, China: A Mixed Methods Study

Xuanchen Tao^1,2^, Limin Mao^3^, Puhong Zhang^1,2^, Xinyan Ma^4^, Zhenyu Liang^4^, Kaige Sun^1^, David Peiris^2^

Affiliations

^1^ The George Institute for Global Health, Beijing, China

^2^ The George Institute for Global Health, Faculty of Medicine and Health, University of New South Wales, Sydney, Australia

^3^ Center for Social Research in Health, University of New South Wales, Sydney, Australia

^4^ Shijiazhuang Center for Disease Control, Shijiazhuang, Hebei Province. China

**Corresponding Authors**

Puhong Zhang

[zpuhong@georgeinstitute.org.cn](mailto:zpuhong@georgeinstitute.org.cn)

Xinyan Ma

[xinyanmacn@hotmail.com](mailto:xinyanmacn@hotmail.com)

**Supplementary Material**

Supplementary 1. Interview Guides

Interview Questions

1. Local Health Administrators:
   1. What is your role? What are your main responsibilities?
   2. What are the main aspects of PHC of chronic disease management currently?
   3. What is the specific workflow for diagnosing and managing chronic disease patients? (Work, information, finances, sources of income, management)
   4. What are the difficulties faced in current chronic disease management? What areas do you think can be improved?
   5. How can village doctors be encouraged to actively use mobile health technology? Any suggestions?
   6. Can family members be involved in long-term chronic disease management? Any suggestions?
   7. How can patients' family members be encouraged to actively participate in this mobile health technology? Any suggestions?
   8. Do you think this mobile health technology can be integrated with the existing health information systems? Will the integrated system meet the requirement of and improve the hierarchical diagnosis and treatment health service system?
   9. Do you think the mobile health technology will have an impact on village doctors' daily patient management? Will it be helpful or hinder their work?
2. Medical Staff
   1. What is your work experience? What are your main job responsibilities?
   2. What is the main work of chronic disease management currently?
   3. What is the specific workflow for diagnosing and managing chronic disease patients? (Work, information, finances, sources of income, management)
   4. How often do patients measure blood sugar, blood pressure, and blood lipid levels (both at home and at the clinic, as well as elsewhere)?
   5. What difficulties are encountered in chronic disease management and what areas do you hope to improve?
   6. What are your experiences with the current system for managing chronic diseases? What aspects are satisfactory and unsatisfactory? What areas do you think can be improved?
   7. Is it feasible to involve patients' family members in the management process through a mobile health platform so that they can communicate with village doctors to jointly manage patients with chronic diseases? How can patients' family members be included in long-term diabetes management?
   8. Do you think it is necessary to communicate with family members regarding chronic disease management? Do you regularly communicate with patients' family members?
   9. Will mHealth technological application facilitate or hinder your daily clinical work?
3. Patients
   1. Briefly describe your chronic disease situation (onset time, medication, medical treatment).
   2. How are you currently managing your diabetes and other chronic diseases? (Medication, monitoring of blood sugar and blood pressure, involvement of family members, frequency of family visits, level of family involvement)
   3. How often do you measure your blood sugar, blood pressure, and blood lipid levels (both at home and at the clinic, as well as elsewhere)?
   4. Do you have any other major health issues? How do you seek medical care when needed? Do you consult with village doctors or go directly to the hospital?
   5. What difficulties do you face in managing your diabetes and other chronic diseases on a daily basis? Are there any areas you believe can be improved?
   6. Are your family members actively involved in helping you manage your diabetes? What difficulties have you encountered? If the family members are not involved, what is the reason?
   7. Do you think it would be feasible to connect your family members with village doctors to jointly manage your diabetes?
   8. Do you think your family members would be willing to use a mobile application to help you manage your diabetes? Why or why not?
4. Family members of patients
   1. Briefly describe the chronic disease situation (onset time, medication, medical treatment) of the patient.
   2. How is the patient at home currently managing their diabetes and other chronic diseases? (Medication, monitoring of blood sugar and blood pressure, involvement of family members, frequency of family visits, level of family involvement)
   3. How often is blood sugar, blood pressure, and blood lipid levels measured (both at home and at the clinic, as well as elsewhere) on a regular basis?
   4. From which sources, do you learn about the current situation of the patients’ diabetes and other chronic diseases?
   5. Are you actively helping the patient at home manage their diabetes and other chronic diseases? (If not, why? What difficulties are encountered? Areas for improvement?)
   6. Is it possible to obtain information about the patient's diabetes and other chronic diseases from the village doctor on a regular basis? (If not, why? What difficulties are encountered?)
   7. What functions do you primarily use on your mobile phone? For example, what are some commonly used apps? What activities do you engage in on your phone? (Do you have the ability/interest to use apps?)
   8. Would you be willing to spend time using such a mobile application to manage your family member's diabetes and other chronic diseases? Why?

Supplementary 2. Thematic Coding Tree

| 1. General Information  1.1 Demographic Characteristics  1.2 Health Literacy  2. Health Policies  2.1 Health Insurances  2.2 Medicine  2.3 Health Examination  2.4 Health Education  2.5 Chronic Disease Management  3. Environment and Resources  3.1 Primary Health Facilities  3.2 Accessibility of Health Facilities  3.3 Diagnostic Equipment  3.3.1 Primary Health Facilities  3.3.2 Patients’ Families  3.4 Management by Information Technology  4. Healthcare Providers  4.1 Quantity  4.2 Background  4.3 Training  4.4 Working Load  4.5 Doctor-Patient Relationship  5. Family Support  5.1 Background  5.2 Family Member - Patient Relationship  5.3 Current Situation  5.4 Willingness to support  5.5 Time and Energy  6. Budget of Primary Health Facilities for Chronic Disease Management  6.1 Funding Sources  6.2 Expenditure  6.3 Staff Income  7. Patients’ Willingness to Healthcare  7.1 Financial Disease Burden  7.2 Non-financial Disease Impact  7.2 Health Expectation  8. Chronic Disease Management Targets  8.1 Current Situation  8.2 Assessment of the National Project on Basic Public Health Services  9. Chronic Disease Management  9.1 New Cases Identification  9.2 Doctor Visiting  9.2.1 PHC facilities  9.2.2 Hospitals  9.3 Follow-up and Monitoring  9.3.1 Monitoring by Doctors  9.3.2 Self-Monitoring  9.4 Drug Therapy  9.5 Non-drug Therapy  10. Chronic Disease Management with mHealth  10.1 Smart Phone Availability  10.2 Access to Internet  10.3 Experience with Mobile Health Management Applications  10.4 Willingness to Use Smart Management |
| --- |

Supplementary 3. Demographic information of all interview participants

| Characteristics  (Mean ± SD or n) | Health Administrators) (N=4) | Medical Staff (N=7) | Patients (N=18) | Family Members (N=13) |
| --- | --- | --- | --- | --- |
| Age (years) | 38 ± 8 | 43 ± 8 | 57 ± 9 | 34 ± 10 |
| Sex |  |  |  |  |
| Male | 2 | 3 | 9 | 5 |
| Female | 2 | 4 | 9 | 8 |
| Highest education level |  |  |  |  |
| Below primary school | 0 | 0 | 4 | 0 |
| Primary school | 0 | 0 | 5 | 1 |
| Middle school | 0 | 0 | 6 | 4 |
| High school | 0 | 0 | 3 | 3 |
| Technical school | 0 | 4 | 0 | 3 |
| Junior college | 1 | 2 | 0 | 1 |
| College | 3 | 1 | 0 | 1 |
| Possession of smart phone | 4 | 7 | 3 | 13 |
| Frequent smart phone internet use | 4 | 7 | 2 | 12 |
| Previous experience with mobile health management applications | 1 | 1 | 1 | 4 |
| Average time of interview (mins) | 46 ± 13 | 57 ± 22 | 22 ± 8 | 18 ± 6 |
